# Characterization of the chloroplast genome of a rare species *Polygonatum* sp. in China (Asparagaceae, Asparagales)

**DOI:** 10.1080/23802359.2020.1720538

**Published:** 2020-02-03

**Authors:** Can Zhong, Jia Lao, Jian Jin, Wei He, Jing Xie, Hao Liu, You Qin, Shuihan Zhang

**Affiliations:** a Institute of Chinese Materia Medica, Hunan Academy of Chinese Medicine, Changsha, PR China;; b Graduate School, Hunan University of Chinese Medicine, Changsha, PR, China;; c Department of Research and Development, Resgreen Group International Inc., Changsha, PR, China

**Keywords:** Polygonatum sp, High-throughput sequencing, Chloroplast, Genome sequence

## Abstract

*Polygonatum* (Asparagaceae) is a medicinal and food plant that is naturally distributed in most of countries throughout the temperate Northern Hemisphere. Here we report on the complete chloroplast (cp) genome sequence of a rare species *Polygonatum* sp. without defoliation in late autumn or winter. The cp genome is 157,696 bp in size and includes two inverted repeat regions of 52,821bp, which is separated by a large single-copy region of 84,359 bp and a small single copy region of 20,516 bp. A total of 132 genes were predicted, including 38 tRNA, 8 rRNA, and 86 protein-coding genes. Phylogenetic analysis placed *Polygonatum* sp. under the subfamily Nolinoideae of the family Asparagaceae.

The genus *Polygonatum* (Asparagaceae, Asparagales) comprises 71 species distributed in China, Korea, Japan, Russia, India, Europe and North America, throughout the temperate Northern Hemisphere (Zhao et al. 2018). The rhizome part is often harvested to be prepared as vegetable or snack that contains nourishing nutrients and medicinal properties (Jin et al. 2019), including polysaccharides, oligosaccharides (Jin et al. 2018) and other bioactive constituents (Hu et al. 2015; Zhou et al. 2015). These *Polygonatum* plants grow in moist and shady places, typically in forests or bushes with thick and fertile soil. The leaves of most of species were fall down in the late autumn, leading to growth stagnation without photosynthesis. Recently, a rare species of *Polygonatum* sp. with no defoliation in late autumn or winter was found in Hunan, China. This *Polygonatum* sp. could be a valuable resource for cultivation.

In this study, we aimed to characterize the complete chloroplast (cp) genome sequence of this *Polygonatum* sp. to serve as a valuable genomic resource for this rare plant species. Total genomic DNA was extracted from fresh leaves of *Polygonatum* sp. planted in Botanical Garden, Institute of Chinese Materia Medica, Hunan Academy of Chinese Medicine (N 28°13′28.15″, E 112°56′26.96″). Additional leaf specimens were kept in Hunan Herbarium of Chinese Traditional Medicine under the collection number HUTM100002.

A genomic library consisting of an insert size of 350 bp was constructed using TruSeq DNA Sample Prep Kit (Illumina, USA) and sequencing was carried out on an Illumina NovaSeq platform. The output was a 6 Gb raw data of 150 bp paired-end reads, further trimmed and assembled using SPAdes (Bankevich et al. 2012). Annotations of cp genome were conducted by the software Geneious (Kearse et al. 2012) and further manually checked by comparison against the *P. verticillatum* complete cp genome (Genbank accession number: KT722981).

The complete cp genome of *Polygonatum* sp. (Genbank accession number: MN906758) is 157, 696 bp in length, displaying a quadripartite structure that contains a pair of inverted repeats (IR) regions (52,821 bp), separated by a large single-copy (LSC) region (84,359 bp) and a small single-copy (SSC) region (20,516 bp). There are 132 genes reported, including 86 protein-coding genes, 8 ribosomal RNA genes, and 38 rRNA genes. The overall GC content of the cp genome was 37.62%.

For phylogenetic analysis, a maximum-likelihood (ML) tree was constructed with 1000 bootstrap replicates using FastTree software (Liu et al. 2011). A subset of 13 species from the family Asparagaceae was included, with 10 species from Liliaceae as outgroup. The ML analysis showed that *Polygonatum* sp. is placed under the subfamily Nolinoideae of the family Asparagaceae, clustered together with other *Polygonatum* species (Figure 1). The taxonomic status of this rare no-defoliation species *Polygonatum* sp. exhibits a closest relationship with *P. sibiricum*, *P. verticillatum* and *P. stenophyllum*. This rare species could be a new species of *Polygonatum*. This finding could serve as valuable genomic resources providing insight into conservation and exploitation efforts for this important plant species.

**Figure 1.**
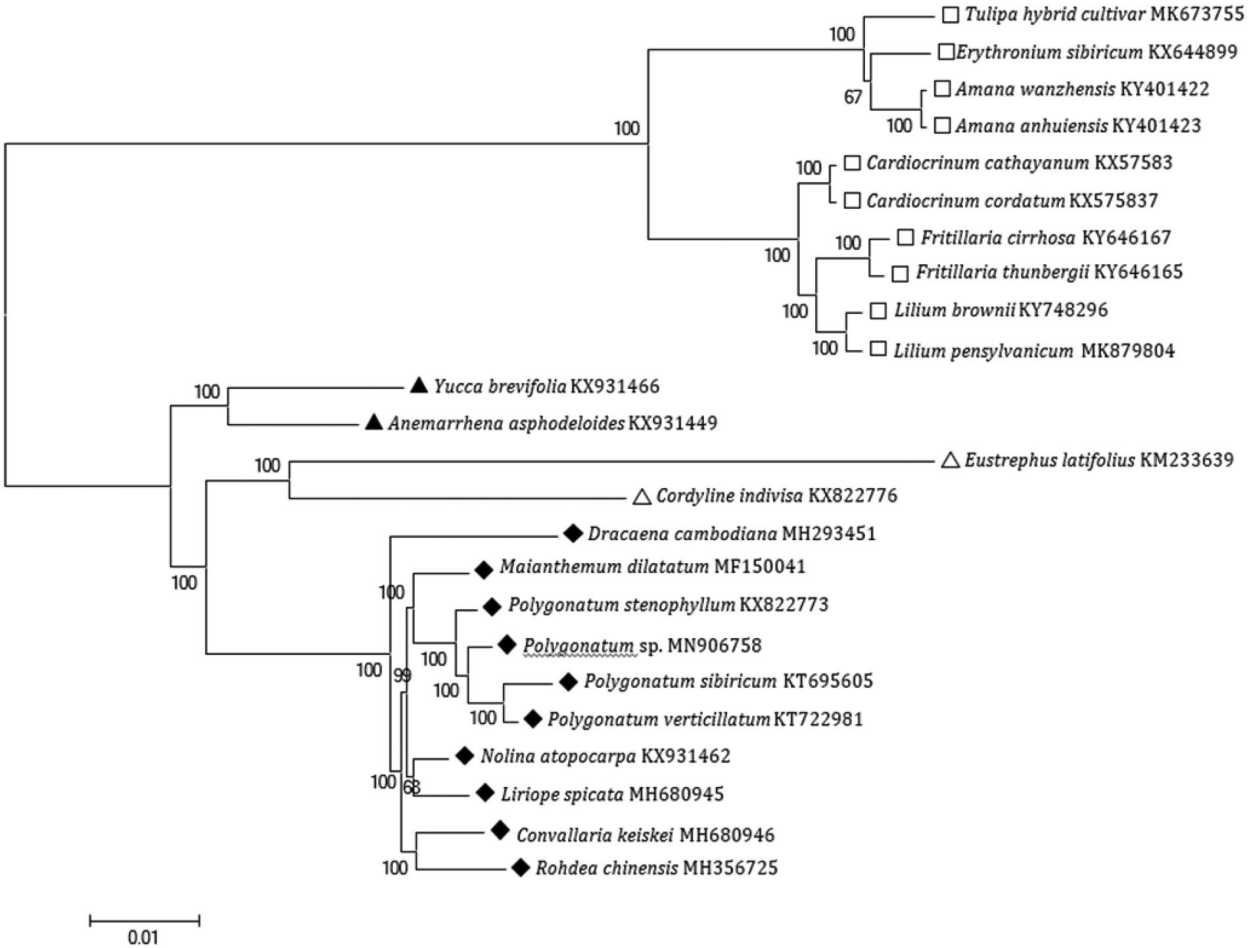
Maximum-likelihood tree based on the complete chloroplast genome sequences of 13 species from the family Asparagaceae with Liliaceae as outgroup. The bootstrap values were based on 1000 replicates.
